# The next-generation Open Targets Platform: reimagined, redesigned, rebuilt

**DOI:** 10.1093/nar/gkac1046

**Published:** 2022-11-18

**Authors:** David Ochoa, Andrew Hercules, Miguel Carmona, Daniel Suveges, Jarrod Baker, Cinzia Malangone, Irene Lopez, Alfredo Miranda, Carlos Cruz-Castillo, Luca Fumis, Manuel Bernal-Llinares, Kirill Tsukanov, Helena Cornu, Konstantinos Tsirigos, Olesya Razuvayevskaya, Annalisa Buniello, Jeremy Schwartzentruber, Mohd Karim, Bruno Ariano, Ricardo Esteban Martinez Osorio, Javier Ferrer, Xiangyu Ge, Sandra Machlitt-Northen, Asier Gonzalez-Uriarte, Shyamasree Saha, Santosh Tirunagari, Chintan Mehta, Juan María Roldán-Romero, Stuart Horswell, Sarah Young, Maya Ghoussaini, David G Hulcoop, Ian Dunham, Ellen M McDonagh

**Affiliations:** Open Targets, Wellcome Genome Campus, Hinxton, Cambridgeshire CB10 1SD, UK; European Molecular Biology Laboratory, European Bioinformatics Institute (EMBL-EBI), Wellcome Genome Campus, Hinxton, Cambridgeshire CB10 1SD, UK; Open Targets, Wellcome Genome Campus, Hinxton, Cambridgeshire CB10 1SD, UK; European Molecular Biology Laboratory, European Bioinformatics Institute (EMBL-EBI), Wellcome Genome Campus, Hinxton, Cambridgeshire CB10 1SD, UK; Open Targets, Wellcome Genome Campus, Hinxton, Cambridgeshire CB10 1SD, UK; European Molecular Biology Laboratory, European Bioinformatics Institute (EMBL-EBI), Wellcome Genome Campus, Hinxton, Cambridgeshire CB10 1SD, UK; Open Targets, Wellcome Genome Campus, Hinxton, Cambridgeshire CB10 1SD, UK; European Molecular Biology Laboratory, European Bioinformatics Institute (EMBL-EBI), Wellcome Genome Campus, Hinxton, Cambridgeshire CB10 1SD, UK; Open Targets, Wellcome Genome Campus, Hinxton, Cambridgeshire CB10 1SD, UK; European Molecular Biology Laboratory, European Bioinformatics Institute (EMBL-EBI), Wellcome Genome Campus, Hinxton, Cambridgeshire CB10 1SD, UK; Open Targets, Wellcome Genome Campus, Hinxton, Cambridgeshire CB10 1SD, UK; European Molecular Biology Laboratory, European Bioinformatics Institute (EMBL-EBI), Wellcome Genome Campus, Hinxton, Cambridgeshire CB10 1SD, UK; Open Targets, Wellcome Genome Campus, Hinxton, Cambridgeshire CB10 1SD, UK; European Molecular Biology Laboratory, European Bioinformatics Institute (EMBL-EBI), Wellcome Genome Campus, Hinxton, Cambridgeshire CB10 1SD, UK; Open Targets, Wellcome Genome Campus, Hinxton, Cambridgeshire CB10 1SD, UK; European Molecular Biology Laboratory, European Bioinformatics Institute (EMBL-EBI), Wellcome Genome Campus, Hinxton, Cambridgeshire CB10 1SD, UK; Open Targets, Wellcome Genome Campus, Hinxton, Cambridgeshire CB10 1SD, UK; European Molecular Biology Laboratory, European Bioinformatics Institute (EMBL-EBI), Wellcome Genome Campus, Hinxton, Cambridgeshire CB10 1SD, UK; Open Targets, Wellcome Genome Campus, Hinxton, Cambridgeshire CB10 1SD, UK; European Molecular Biology Laboratory, European Bioinformatics Institute (EMBL-EBI), Wellcome Genome Campus, Hinxton, Cambridgeshire CB10 1SD, UK; Open Targets, Wellcome Genome Campus, Hinxton, Cambridgeshire CB10 1SD, UK; European Molecular Biology Laboratory, European Bioinformatics Institute (EMBL-EBI), Wellcome Genome Campus, Hinxton, Cambridgeshire CB10 1SD, UK; Open Targets, Wellcome Genome Campus, Hinxton, Cambridgeshire CB10 1SD, UK; European Molecular Biology Laboratory, European Bioinformatics Institute (EMBL-EBI), Wellcome Genome Campus, Hinxton, Cambridgeshire CB10 1SD, UK; Open Targets, Wellcome Genome Campus, Hinxton, Cambridgeshire CB10 1SD, UK; European Molecular Biology Laboratory, European Bioinformatics Institute (EMBL-EBI), Wellcome Genome Campus, Hinxton, Cambridgeshire CB10 1SD, UK; Open Targets, Wellcome Genome Campus, Hinxton, Cambridgeshire CB10 1SD, UK; European Molecular Biology Laboratory, European Bioinformatics Institute (EMBL-EBI), Wellcome Genome Campus, Hinxton, Cambridgeshire CB10 1SD, UK; Open Targets, Wellcome Genome Campus, Hinxton, Cambridgeshire CB10 1SD, UK; European Molecular Biology Laboratory, European Bioinformatics Institute (EMBL-EBI), Wellcome Genome Campus, Hinxton, Cambridgeshire CB10 1SD, UK; Open Targets, Wellcome Genome Campus, Hinxton, Cambridgeshire CB10 1SD, UK; European Molecular Biology Laboratory, European Bioinformatics Institute (EMBL-EBI), Wellcome Genome Campus, Hinxton, Cambridgeshire CB10 1SD, UK; Open Targets, Wellcome Genome Campus, Hinxton, Cambridgeshire CB10 1SD, UK; Wellcome Sanger Institute, Wellcome Genome Campus, Hinxton, Cambridgeshire CB10 1SA, UK; Open Targets, Wellcome Genome Campus, Hinxton, Cambridgeshire CB10 1SD, UK; Wellcome Sanger Institute, Wellcome Genome Campus, Hinxton, Cambridgeshire CB10 1SA, UK; Open Targets, Wellcome Genome Campus, Hinxton, Cambridgeshire CB10 1SD, UK; Wellcome Sanger Institute, Wellcome Genome Campus, Hinxton, Cambridgeshire CB10 1SA, UK; Open Targets, Wellcome Genome Campus, Hinxton, Cambridgeshire CB10 1SD, UK; European Molecular Biology Laboratory, European Bioinformatics Institute (EMBL-EBI), Wellcome Genome Campus, Hinxton, Cambridgeshire CB10 1SD, UK; Open Targets, Wellcome Genome Campus, Hinxton, Cambridgeshire CB10 1SD, UK; European Molecular Biology Laboratory, European Bioinformatics Institute (EMBL-EBI), Wellcome Genome Campus, Hinxton, Cambridgeshire CB10 1SD, UK; Open Targets, Wellcome Genome Campus, Hinxton, Cambridgeshire CB10 1SD, UK; Wellcome Sanger Institute, Wellcome Genome Campus, Hinxton, Cambridgeshire CB10 1SA, UK; GlaxoSmithKline plc, GSK Medicines Research Centre, Gunnels Wood Road, Stevenage, SG1 2NY, UK; Open Targets, Wellcome Genome Campus, Hinxton, Cambridgeshire CB10 1SD, UK; European Molecular Biology Laboratory, European Bioinformatics Institute (EMBL-EBI), Wellcome Genome Campus, Hinxton, Cambridgeshire CB10 1SD, UK; European Molecular Biology Laboratory, European Bioinformatics Institute (EMBL-EBI), Wellcome Genome Campus, Hinxton, Cambridgeshire CB10 1SD, UK; European Molecular Biology Laboratory, European Bioinformatics Institute (EMBL-EBI), Wellcome Genome Campus, Hinxton, Cambridgeshire CB10 1SD, UK; Open Targets, Wellcome Genome Campus, Hinxton, Cambridgeshire CB10 1SD, UK; European Molecular Biology Laboratory, European Bioinformatics Institute (EMBL-EBI), Wellcome Genome Campus, Hinxton, Cambridgeshire CB10 1SD, UK; Open Targets, Wellcome Genome Campus, Hinxton, Cambridgeshire CB10 1SD, UK; European Molecular Biology Laboratory, European Bioinformatics Institute (EMBL-EBI), Wellcome Genome Campus, Hinxton, Cambridgeshire CB10 1SD, UK; Open Targets, Wellcome Genome Campus, Hinxton, Cambridgeshire CB10 1SD, UK; Wellcome Sanger Institute, Wellcome Genome Campus, Hinxton, Cambridgeshire CB10 1SA, UK; Open Targets, Wellcome Genome Campus, Hinxton, Cambridgeshire CB10 1SD, UK; Wellcome Sanger Institute, Wellcome Genome Campus, Hinxton, Cambridgeshire CB10 1SA, UK; Open Targets, Wellcome Genome Campus, Hinxton, Cambridgeshire CB10 1SD, UK; Wellcome Sanger Institute, Wellcome Genome Campus, Hinxton, Cambridgeshire CB10 1SA, UK; Open Targets, Wellcome Genome Campus, Hinxton, Cambridgeshire CB10 1SD, UK; GlaxoSmithKline plc, GSK Medicines Research Centre, Gunnels Wood Road, Stevenage, SG1 2NY, UK; Open Targets, Wellcome Genome Campus, Hinxton, Cambridgeshire CB10 1SD, UK; European Molecular Biology Laboratory, European Bioinformatics Institute (EMBL-EBI), Wellcome Genome Campus, Hinxton, Cambridgeshire CB10 1SD, UK; Wellcome Sanger Institute, Wellcome Genome Campus, Hinxton, Cambridgeshire CB10 1SA, UK; Open Targets, Wellcome Genome Campus, Hinxton, Cambridgeshire CB10 1SD, UK; European Molecular Biology Laboratory, European Bioinformatics Institute (EMBL-EBI), Wellcome Genome Campus, Hinxton, Cambridgeshire CB10 1SD, UK

## Abstract

The Open Targets Platform (https://platform.opentargets.org/) is an open source resource to systematically assist drug target identification and prioritisation using publicly available data. Since our last update, we have reimagined, redesigned, and rebuilt the Platform in order to streamline data integration and harmonisation, expand the ways in which users can explore the data, and improve the user experience. The gene–disease causal evidence has been enhanced and expanded to better capture disease causality across rare, common, and somatic diseases. For target and drug annotations, we have incorporated new features that help assess target safety and tractability, including genetic constraint, PROTACtability assessments, and AlphaFold structure predictions. We have also introduced new machine learning applications for knowledge extraction from the published literature, clinical trial information, and drug labels. The new technologies and frameworks introduced since the last update will ease the introduction of new features and the creation of separate instances of the Platform adapted to user requirements. Our new Community forum, expanded training materials, and outreach programme support our users in a range of use cases.

## INTRODUCTION

The Open Targets consortium brings together expertise and capabilities from academic and pharmaceutical industry partners with the vision to systematically identify targets that will ultimately lead to more effective and safer drugs for disease treatment. To facilitate therapeutic hypothesis building, the consortium experimentally generates novel evidence and contextualises it with available knowledge in the public domain. In this task, genetics provides an unprecedented source of causal evidence, to the extent that two-thirds of the drugs approved in 2021 were directly or indirectly supported by genetic evidence (1–3).

**Figure 1.**
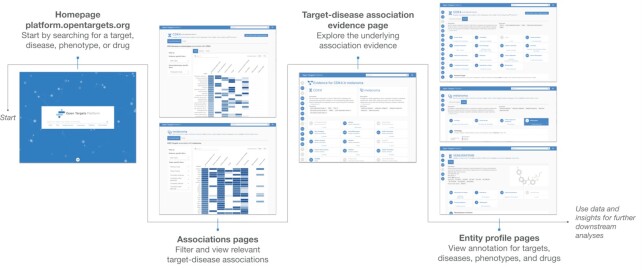
A journey through the Platform web interface. The Open Targets Platform web interface is the first point of access for most users, and was completely redesigned to create the Next Generation Platform. The unified search box is connected to a series of tools allowing users to query different therapeutic hypotheses. From the homepage, users can navigate to association pages, with prioritised lists of target–disease associations. From there, users can access target–disease evidence pages, detailing the available evidence for an association. Once the evidence for a target–disease association has been assessed, users can explore entity profile pages, containing annotation information for each target, disease/phenotype and drug in the Platform to further build their hypothesis. For targets, this includes investigating whether it is expressed in a suitable tissue, what type of modality may be suitable and whether modulation is likely to be safe, whether there are already known drugs or available chemical probes for validation experiments, and whether interacting proteins may be more suitable targets. For disease/phenotype, the user can investigate known drugs and their targets, or explore targets associated with disease phenotypes through ontology expansion. Drug annotation pages provide a user with the mechanism of action and safety information related to modulating a target.

The Open Targets Platform (https://platform.opentargets.org/) provides an open source informatic solution for the identification and prioritisation of targets using publicly available data (4). The Platform provides the necessary knowledgebase to characterise targets, diseases and drugs in the context of drug discovery, as well as the relationships between the entities, with particular focus on target–disease associations (Figure 1). Powered with an in-house target identification scoring framework, evidence is aggregated across sources to provide ranked lists of gene–disease associations. To maintain up-to-date-evidence, ensure regular updates from external data sources and integrate user feedback and new features, the Platform is released five times a year.

Within the last 2 years, the Platform has been widely used by the community as a source of truth in various contexts, including; supporting Crohn's disease associations or approved kinase inhibitors, as a prioritisation tool in the search for drug repurposing opportunities, or more generally as a data source to build corporate knowledge graphs to assist drug development (5–7). Moreover, our open-source code has been re-used by the NIH National Cancer Institute Childhood Cancer Data Initiative to develop and launch a customised Molecular Targets Platform that integrates patient-specific data, aiming to facilitate therapeutic hypotheses building and target discovery in paediatric cancer (https://moleculartargets.ccdi.cancer.gov/). This is just the first example of the many potential applications of the Platform open-source code to create separate instances in order to integrate experimental or pre-publication results.

**Figure 2.**
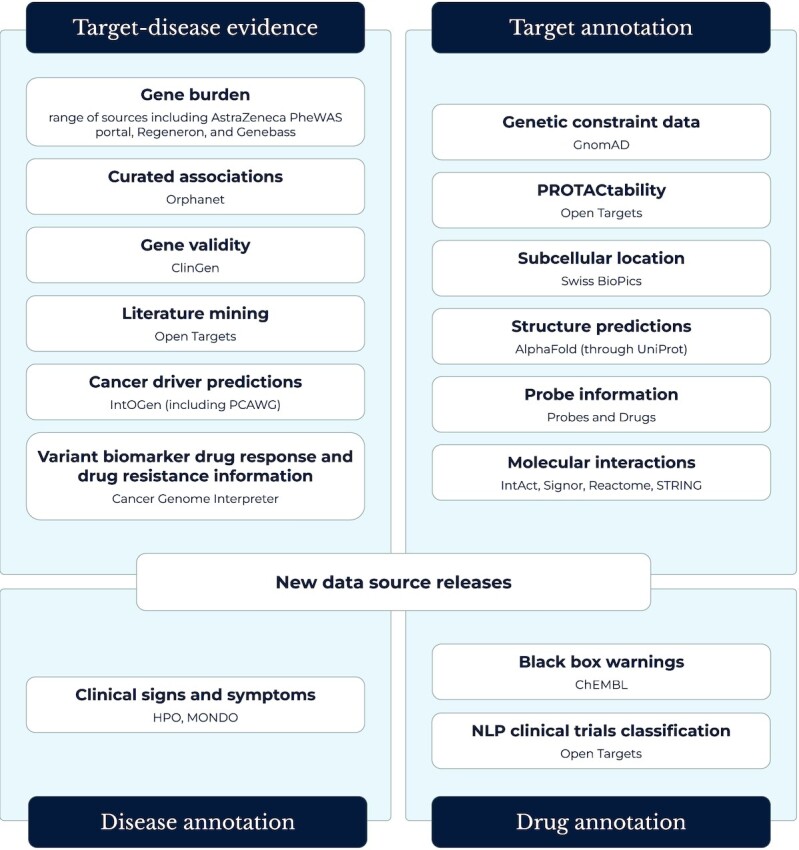
New data in the Open Targets Platform. Additional sources of target–disease evidence and new types of annotation for targets diseases and drugs during the last 2 years.

Herein we provide an update on the significant enhancements made to the Platform since our last publication that contribute to supporting the aims of the consortium as well as the world-wide community (Figure 2).

### Enhanced target–disease evidence

The Platform assists target identification by integrating multiple layers of information pinpointing likely causal targets with biological plausibility. A fundamental element of this effort is the target–disease evidence, which we collate from 22 different sources, covering evidence derived from genetic associations, somatic mutations, known drugs, differential expression, animal models, pathways and systems biology. Since the last update, we have expanded the Platform to include several new data sources which we describe below, as well as providing regular updates and expanding the available information on each piece of evidence. Some of the data streams stem directly from public resources in our partner institutions (8–14). An important aspect of the data integration is that all the phenotypic traits are mapped using the Experimental Factor Ontology (EFO), which we have recently expanded to better cover quantitative traits and medical procedures (15).

#### Common and complex diseases

For common variation, the Platform uniquely relies on the closely developed post-GWAS analysis available in Open Targets Genetics (https://genetics.opentargets.org/) (16). Since the last update, the Genetics portal has integrated new studies deposited in the GWAS Catalog—with or without summary statistics—as well as a series of FinnGen public data releases (17,18). To enhance the causal inference, the Genetics portal enriched the L2G machine learning (ML) regression with additional eQTLs from GTEx v8 and pQTLs from 6 new studies (19,20).

#### Gene burden analysis

**Figure 3.**
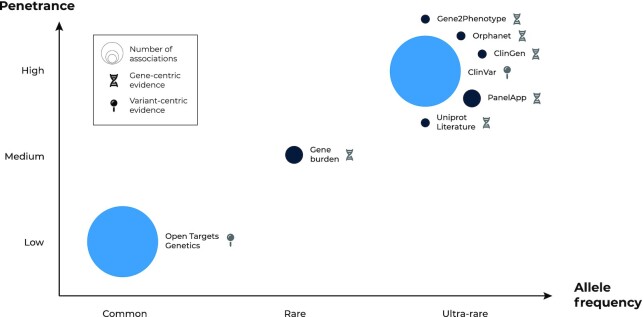
Schematic representation of germline genetic evidence in the Platform. Data sources—bubbles—are classified based on the predominant allelic frequency and penetrance of the reported genetic variation. The size of the bubbles represents the number of target–disease associations provided by each data source sorted into three bins: 1) 1000–10 000—from 1966 (ClinGen) to 8506 (Orphanet); 2) 10 000–100 000—with 27 162 target–disease associations from Gene Burden analyses to 40 446 from PanelApp; 3) and 100 000 + associations (Open Targets Genetics: 694 214; ClinVar: 1 541 903). Data sources are classified depending on whether they capture genetic variation at the variant (lighter blue) or gene level (dark blue).

The increasing number of sequenced individuals has led to a proliferation of studies aimed at identifying rare or ultra-rare variants in coding regions that contribute to common diseases. To respond to this new source of target–disease evidence, we included a new Gene Burden dataset capturing the results from collapsing analysis on exome or whole-genome sequencing studies (Figure 3). Among other publications, we ingested three studies analysing the 450 000 exomes sequenced by the UK BioBank to a total of 5459 unique gene–disease associations, 57% of them not previously reported on any other genetic resource included in the Platform (21–23). The aggregation of likely deleterious variants in coding regions also provides a putative mechanistic explanation that can be leveraged to build a therapeutic hypothesis. Moreover, gene burden constitutes the first ancestry-specific evidence in the Platform, a feature that will be expanded to other data sources where possible.

#### Rare diseases

The Platform continues to integrate several resources providing variant- and gene-centric evidence derived from the clinical assessment of families or individuals with rare diseases. Since the last update, we included new versions of these resources mapped to EFO and two new datasets: Orphanet and the ClinGen Gene-Disease Validity assessments which provide an evaluation of the strength of the evidence supporting or refuting a claim that variation in a particular gene causes a particular disease (24,25). Each respective resource currently brings 6003 and 1655 unique associations, with 19.3% and 28.7% unique target–disease pairs not captured by any other genetic resource in the Platform. To align with the Gene Curation Coalition guidelines, we have adopted the standardised terminologies for confidence level and display allelic requirements when available (26). In collaboration with the European Variation Archive, we have also included complex structural variants in ClinVar such as insertions, deletions, and small tandem repeats, aiming to cover a broader range of disease-causing mechanisms (27).

#### Somatic variation

A new data widget was created to bring in cancer biomarker annotations sourced from the Cancer Genome Interpreter, which provide evidence for target–disease associations (and importantly the level of clinical evidence from pre-clinical to approved drug standard-of-care guidelines) (28). These also contribute to biomarker-drug response and drug resistance information. We also brought in cancer driver predictions from IntOgen's analysis of the Pan-Cancer Analysis of Whole Genomes study of the International Cancer Genome Consortium, and developed the visualisation to provide clear information of the different driver methods used to predict the target as a driver gene in different cohorts (29).

### New literature mining strategy

The Platform relies on knowledge extraction from the biomedical literature as an additional source of target–disease evidence and annotation for the three main entities: targets, diseases, and drugs. In collaboration with Europe PubMedCentral, we refactored the pipeline to perform named-entity recognition on all abstracts and open access full-text documents using a BioBERT model fine-tuned for the task (9,30). To normalise the identified words to the Platform entities, we leverage identifiers, names, symbols and synonyms from diverse sources. In line with the previous implementation, once the entity occurrences are associated with up-to-date identifiers the new pipeline generates evidence based on sentences in which target and disease co-occurred (31). The bibliography widget allows users to interrogate the literature by entity, displaying a list of publications mentioning the selected term. The user can also expand the selection by including other suggested entities in the search based on the similarity of their BioBERT-driven descriptors to the previously selected terms.

The development and integration of natural language processing (NLP) models into the Platform pipelines have enabled additional applications. We now provide a classification of the reasons why a clinical trial stopped—more significantly, separating studies stopped due to efficacy or side effects from those halted due to other reasons independent of the therapeutic hypothesis. In parallel, a ML model is used to expand the list of drug indications by mining drug labels (11). Overall, the expansion of NLP applications allows the Platform to provide insights into semi-structured data relevant to building a more robust therapeutic hypothesis.

### Improving target characterisation

Prioritising likely causal targets in a drug discovery program requires a comprehensive understanding of the target biology that could inform risks and strategies for modulating the target. Since the last update, several enhancements aim to expand the Platform's ability to inform about the target tractability for different modalities. For example, the PROTAC tractability assessment includes a set of target properties that can help determine whether protein degradation would be a suitable modulation strategy (32). Similarly, we expanded the list of tool molecules that are selective and specific against the target by including the data integration from Probes and Drugs (33). The recent inclusion of the AlphaFold protein structure predictions in the target profile page allows the identification of potential druggable pockets in previously unresolved 3D models (34). This information, combined with the enhanced subcellular localisation widget, better informs about the most likely viable target modulation strategy (35).

Identifying target properties that might raise safety issues can also influence the prioritisation strategy. A new ‘Genetic Constraint’ data widget aims to capture how evolutionarily protected a gene is from loss-of-function variation, a proxy to inform whether modulating the target is likely to be tolerated. This section includes the observed and expected frequency of synonymous, nonsynonymous, and predicted loss-of-function mutations together with the pLOEUF genetic constraint assessment provided by GnomAD (36). In addition, the safety widget information has been expanded to incorporate further safety events associated with targets. On drug profile pages, BlackBox warnings and drug withdrawal information have also been expanded to increase the drug-associated safety events (37).

To contextualise the target in the context of its community, we incorporated the new molecular interactions section (38). This feature aggregates and integrates the network topology, as well as the supporting evidence from 4 different sources capturing different types of interactions; IntAct (physical interactions), STRING (functional interactions), Signor (directional signalling interactions), and Reactome (enzymatic reaction pathway-based interactions) (14,39–41). The data widget for molecular interactions allows the user to expand upon their therapeutic hypothesis and explore options for alternative targets that interact with or share the same biological pathway with a known disease-causing protein or a protein for which there is already a known drug.

### The next-generation platform

Since the last update, the Open Targets Platform underwent a major refactor of all its data and services in an attempt to unblock new target identification and prioritisation capabilities. Powered by a streamlined data model, the newly developed Spark extract-transform-load pipeline produces a set of datasets capturing all of the Platform knowledgebase. Notably, the target–disease evidence dataset now integrates potentially causal evidence from our 22 datasources in a harmonised format, simplifying the development of any downstream applications. Similarly, a newly designed GraphQL API serves the information to a brand new React web application that was released to the public in June 2021.

While the web application remains the Platform entry point for most users (Figure 1), a larger range of options exists for power users of the data. The GraphQL API might be a good option for users planning to query a few records in the data, with the downloadable datasets representing the most interesting option for most users with some programming experience. For cloud developers, the Platform data was included in 2022 as a public dataset in Google BigQuery and AWS Open Data. A comprehensive description of the Platform data and how to access it can be found in the renovated documentation site (https://platform-docs.opentargets.org).

### The Open Targets community

The expansion of the Open Targets Platform was paralleled by an expansion of our outreach activities. In particular, we created the Open Targets Community forum (community.opentargets.org) in April 2021, to host discussions, foster collaborations, and leverage feedback from our users. We also developed our training materials to support users in a variety of mediums and access levels. For example, working with EMBL-EBI’s training team, we hosted a series of webinars, which were repurposed intro training courses on the Platform and its API (https://www.ebi.ac.uk/training/online/courses/open-targets-quick-tour/). We also featured posts on the Open Targets blog detailing how to access and use the data in the Platform, the architecture of our re-written pipeline, and our plans for the future of the Platform.

## DISCUSSION

Target identification and prioritisation remain fundamental challenges in the goal of developing safe and effective medicines. As a core resource of the Open Targets consortium, the Platform aims to capitalise upon the expertise and data produced in the public–private partnership, as well as from the public realm, to provide a systematic resource in the pre-competitive target discovery space. The continuous generation of potential causal evidence from increasingly complicated data models, granularity of disease characterisation, and large population-scale datasets, forced us to redesign the core functionalities of the Platform leading to the more modern resource described here. The adoption of these new technologies and frameworks will speed up future development, enhancing the open source contributions to the codebase (https://github.com/opentargets), and facilitate the creation of separate instances of the Platform. It will allow us to bring in more complex datasets (such as temporal single cell transcriptomics) and introduce sophisticated ways for users to build therapeutic hypotheses.

## DATA AVAILABILITY

All code is available in GitHub: https://github.com/opentargets.

## Notes

Present address: Ellen M. McDonagh, Open Targets and European Molecular Biology Laboratory - European Bioinformatics Institute (EMBL-EBI), Wellcome Genome Campus, Hinxton, Cambridgeshire CB10 1SD, UK.

Present address: David Ochoa, Open Targets and European Molecular Biology Laboratory - European Bioinformatics Institute (EMBL-EBI), Wellcome Genome Campus, Hinxton, Cambridgeshire CB10 1SD, UK.
